# The effectiveness and safety of Tai Chi for patients with essential hypertension: study protocol for an open-label single-center randomized controlled trial

**DOI:** 10.1186/s12906-020-03192-z

**Published:** 2021-01-07

**Authors:** Yuxi Li, Dongling Zhong, Chao Dong, Lihong Shi, Yaling Zheng, Yongguo Liu, Qiaoqin Li, Hui Zheng, Juan Li, Tianyu Liu, Rongjiang Jin

**Affiliations:** 1 School of Acupuncture-Moxibustion and Tuina, Chengdu University of Traditional Chinese Medicine, Chengdu, China; 2 School of Health Preservation and Rehabilitation, Chengdu University of Traditional Chinese Medicine, Chengdu, China; 3 The General Hospital of Western Theater Command, Chengdu, China; 4 Knowledge and Data Engineering Laboratory of Chinese Medicine, School of Information and Software Engineering, University of Electronic Science and Technology of China, Chengdu, China; 5 School of Sports, Chengdu University of Traditional Chinese Medicine, Chengdu, China

**Keywords:** Hypertension, Tai Chi, Open label, Randomized controlled trials, Protocol

## Abstract

**Background:**

Evidence showed that Tai Chi may have beneficial effects among hypertensive individuals, although the results are not convincing. We aim to conduct a high-quality clinical trial with 24-h BP measurement to provide robust evidence of Tai Chi for essential hypertension.

**Methods:**

This is an open-label single-center randomized controlled trial with 3 parallel arms. We will compare Tai Chi with walking and waiting-list control. We will recruit 234 hypertensive patients with mild to moderate essential hypertension and randomly assign them to 3 different groups. Participants in Tai Chi group will receive a group-format Yang style 24-form Tai Chi exercise program, 3 sessions per week for 12 weeks. The walking group will be asked to walk, 3 sessions per week for 12 weeks. The waiting-list group will not receive any interventions and/or exercise training. The primary outcome is the change in average 24-h systolic blood pressure (SBP) between baseline and 12 weeks after randomization. The secondary outcomes include 24-h Diastolic Blood Pressure (DBP), average SBP and average DBP during the daytime and night-time, blood pressure (BP) variability, SBP load and DBP load, circadian rhythm of BP, and morning BP surge, endothelial functional indicators, home measured BP, quality of life, adverse events and so on.

**Discussion:**

We expect findings of this trial will provide important insight into application of Tai Chi as an effective and acceptable method for hypertensive patients. Successful completion of this proposed study will also contribute to promotion of Tai Chi in the community in the future.

**Trial registration:**

Clinicaltrials.gov registry: https://clinicaltrials.gov/ct2/show/NCT04267471, date: February 12, 2020.

## Background

Hypertension is the most common risk factor of stroke and cardiovascular diseases, which accounted for 9.4 million deaths worldwide every year [1, 2]. The vast majority of instances of high blood pressure pertains to essential hypertension, which is the presence of high blood pressure while there is no detectable medical or organic cause. According to the World Health Organization report, hypertension affects 22% of adult population around the world [3], and studies showed that the number of hypertensive patients was still increasing [4]. A national wide hypertension survey from 2012 to 2015 reported that the prevalence of hypertension among Chinese population was 23.2% (estimated 244.5 millions people) [5]. Even so, the control rate of hypertension is quite poor. A cross-sectional study of 57,840 hypertensive patients from 17 countries showed that the awareness rate of hypertension was 46.5%, merely 41% received treatment, and only 13.2% had blood pressure (BP) under controll [6]. Pharmacological therapies are the mainstream treatment for hypertension. However, Cochrane reviews showed that many patients discontinued treatment due to undesirable adverse effects [7, 8].

Evidence showed that regular physical exercise had protective effects on hypertensive individuals [9]. Aerobic activity has been recommended to hypertensive patients by Canadian Hypertension Education Program (CHEP), Eighth Joint National Committee (JNC-8), and American Heart Association (AHA) [10–12]. Tai Chi, a traditional mind-body Chinese exercise, is an aerobic exercise with moderate exercise intensity [13]. It combines deep-breath relaxation and gentle movements in sequence with mind concentration. A number of clinical trials reported that Tai Chi not only had positive effects in healthy population [14–17], it could also improve people’s pathological conditions, including Parkinson’s disease [18], fibromyalgia [19] and knee osteoarthritis [20].

Several clinical studies reported that Tai Chi may have some benefits in reducing BP and improving quality of life in hypertensive patients [21–27]. We conducted a systematic review of the existing trials of Tai Chi for essential hypertension [28]. However, we found high heterogeneity and poor methodological quality in these studies, which decreased the liability of evidence. Therefore, we will conduct this randomized controlled trial (RCT) aiming at: first, confirming whether Tai Chi is efficacious for patients with essential hypertension; second, observing the short- and long-term effect of Tai Chi; third, investigating the influence of Tai Chi on endothelial function. In addition, we intend to use 24-h BP measurement to monitor patients’ BP, so as to observe the changes more accurately. In order to provide high quality of evidence, we will conduct this trial in line with Standard Protocol Items: Recommendations for Interventional Trials (SPIRIT) [29] and report our findings in accordance with the Consolidated Standards of Reporting Trial (CONSORT) statement [30]. This clinical trial is registered with an identifier (NCT04267471) at clinicaltrials.gov in February 2020.

## Methods/design

### Design and setting

**Fig. 1:**
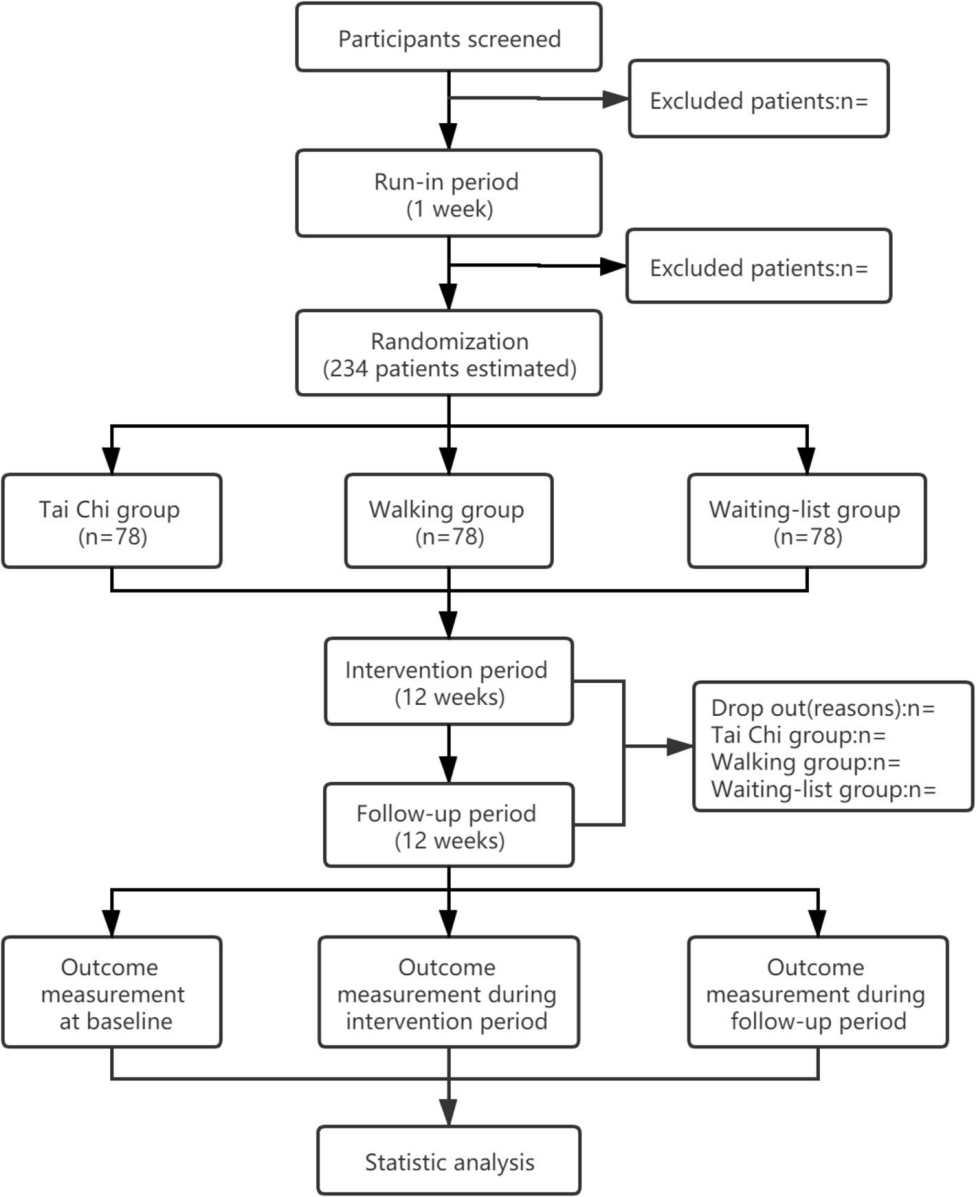
Flow diagram of study design

This is an open-label single-center RCT with 3 parallel arms. This trial has been approved by the regional ethical review committee of traditional Chinese medicine in Sichuan Province in March 2019 (reference number: 2019KL-001). We will compare Tai Chi with walking and waiting-list control. The trial will be conducted in the Hospital of Chengdu University of Traditional Chinese Medicine. The whole study period is 25 weeks, with 1-week baseline, 12-week intervention phase and 12-week follow-up phase. After randomization, participants in Tai Chi group and walking group will receive exercise training 3 times per week for continuous 12 weeks. Participants in waiting-list group can decide whether or not to receive exercise training after follow-up period. Outcomes will be measured at baseline, 12 weeks and 24 weeks after randomization. All participants will be required to sign the written informed consent before randomization. This protocol was compiled in line with the SPIRIT 2013 [29] (Table 1). The flow diagram of this trial is presented in Fig. 1.

**Table 1:**
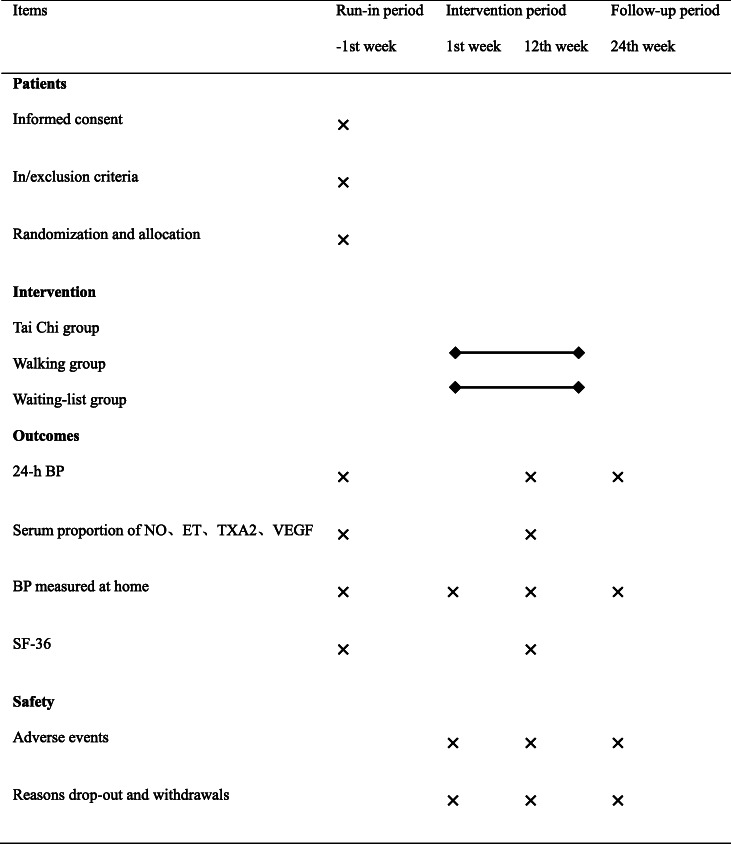
A standard protocol items: recommendation for interventions for trials (SPIRIT)

*BP* Blood pressure, *NO* Nitric Oxide, *ET* Endothelin, *TXA2* Thromboxane A2, *VEGF* Vascular endothelial growth factor, *SF-36* Medical Outcomes Study 36-Item Short Form

### Participants

#### Sample size

This study is an RCT, the intervention group is the Tai Chi group, and the control groups are walking group and waiting-list group. The reduction of Systolic Blood Pressure (SBP) is set as the primary outcome. According to previous study [31], the mean difference and standard deviation of SBP was − 13.33 ± 46.73, − 12.46 ± 33.37 and 3.37 ± 46.01 for Tai Chi compared with walking and waiting-list control. The sample size was calculated using One-Way analysis of variance, F-Tests with the PASS 15.0 software. The standard deviation is set as 20. A total of 65 participants per arm are needed to achieve a power of 80% at a significant level of 5%. A sample size of 195 participants is required to sufficiently detect a target effect size. Considering a 20% drop-out rate, we intend to enroll a total of 234 participants, with 78 participants in each group.

#### Recruitment

Participants will be recruited by posters, doctor’s recommendation, internet and WeChat in Chengdu city. If participants meet the study criteria, they will be required to sign informed consent form prior to inclusion of the study.

#### Diagnostic criteria

According to the 2010 Chinese Guidelines for the Management of Hypertension [32], patients with SBP ≥ 140 mmHg and/or Diastolic Blood Pressure (DBP) ≥ 90 mmHg without using antihypertensive drugs for 3 times on different days are diagnosed with hypertension. The patient who has a history of hypertension and is currently using antihypertensive drugs with BP below 140/90 mmHg is also diagnosed with hypertension. All patients will be diagnosed by 2–3 cardiovascular physicians with more than 10 years of experience.

#### Inclusion criteria

Patients who fulfill all the following inclusion criteria will be included in this study:

1. aged between 45 to 80 years;
2. diagnosed with mild to moderate essential hypertension, and meet the diagnostic criteria of mild to moderate essential hypertension according to 2010 Chinese Guidelines for the Management of Hypertension (140 mmHg≤SBP ≤ 169 mmHg and/or 90 mmHg≤DBP ≤ 109 mmHg) [31];
3. with or without antihypertensive medication;
4. with no regular exercise in the past 3 months;
5. willing to comply with the study protocol;
6. willing to sign informed consent form.

#### Exclusion criteria

Patients with one of the following criteria will be excluded:

1. diagnosed with secondary hypertension or refractory hypertension [33];
2. with severe medical visceral condition and chronic diseases, such as diabetes, epilepsy, severe depression or anxiety, psychosis;
3. with severe bone and joint diseases or motor dysfunction limit ability to participant exercise;
4. with severe cognitive decline (Montreal Cognitive Assessment, MoCA [34], < 26)
5. with decreased myodynamia, poor balance or limited vision that would impede full participation in the study;
6. participated in other clinical trials at the same time.

### Allocation and randomization

Included participants will be randomly assigned to 3 groups (Tai Chi group, walking group, and waiting-list group) with 1:1:1 ratio. The randomization sequence will be created using R 3.5.1 by an independent statistician. The information of random numbers, allocation and intervention will be packed in opaque envelopes, and will be concealed to screeners and outcome assessors. A research assistant will inform eligible participants the allocation results through phone call after opening envelopes in order.

### Blinding

Due to the nature of Tai Chi, participants and researchers are impossible to be blinded to the group assignment. To minimize bias, all outcomes will be measured at baseline, 12 weeks and 24 weeks after randomization by the same independent experienced assessors who are blinded to the allocation. After completing the statistical analyses, the group assignment will be revealed by the project manager.

### Withdrawal criteria and management

Participants will be allowed or required to withdraw from the trial if:

1. participants developing a serious disease which is not suitable to continue in the investigator’s opinion;
2. seriously adverse events happen;
3. participants request to withdraw from the trial.

The reasons and exit time will be recorded in standard case report forms (CRF).

### Interventions

All participants will be asked to maintain usual diet and lifestyle. We will use CRF to record session attendance of participants in Tai Chi group and walking group. In order to monitor the progress, we will collect and analyze the session attendance as well as the self-reported diaries.

#### Tai Chi exercise

Participants in Tai Chi group will be firstly instructed Yang style 24-form Tai Chi by the coaches with at least 5 years of teaching experience during the run-in period. After 3 days of teaching, coaches will assess the qualification of each participants based on: (1) accuracy of movement; (2) coordination and continuity of movement; (3) pauses times. Participants whose fluency and accuracy of Tai Chi movement reach over 60% with less than 5 times of pauses will be considered to be qualified. Unqualified patients will continue a 3-day teaching and a secondary evaluation. During the intervention period, qualified participants will receive a group-format exercise program, 3 sessions per week for 12 weeks. Each session will last 60 min, including a 10-min warm-up, a 40-min Tai Chi practice and a 10-min cool-down.

#### Walking exercise

Using aerobic exercise to reduces BP has been confirmed [35, 36] and recommended in hypertension management guidelines [32, 37]. Walking is a simple and easy aerobic exercise for the elderly to do. We will use walking with targeted intensity as an active controlled. Walking group will be asked to walk, 3 sessions per week for 12 weeks. Each session will last 60 min, including a 10-min warm-up, a 40-min walking and a 10-min cool-down. To ensure the compliance of exercise, participants will be asked to record the start and end time of each exercise session in self-reported diaries.

#### Exercise intensity of Tai Chi and walking

Studies showed that Tai Chi is an aerobic exercise with moderate intensity [13, 38, 39]. In order to achieve the comparability of Tai Chi group and walking group, we will use fitness tracker (HUAWEI HONOR 3, HUAWEI TECHNOLOGIES CO., LTD., China) to record participants’ heart rate during exercises to estimate exercise intensity in accordance with the definition of American College of Sports Medicine of moderate intensity aerobic exercise [40]. The target intensity is 64–76% of maximal heart rate (220-the person’s age). To ensure that participants in Tai Chi group and walking group exercised with the intended intensity throughout the training period, exercise intensity will be calculated and adjusted for 3 times during intervention periods (between weeks 1 to 4, 4 to 8, and 8 to 12).

#### Waiting list

Participants in waiting-list group will not receive any interventions and/or exercise training during the intervention period. They will give an option to receive Tai Chi training after follow-up period.

### Baseline assessment

The participants will undergo baseline assessment before intervention. A descriptive exploratory questionnaire will be administered to collect information as follows: (1) demographic and socioeconomic variables (gender, age, socioeconomic status, and education); (2) behavioral variables (physical activity, smoking, and alcohol consumption); (3) anthropometric variables (bodyweight, height, and waist circumference); (4) clinical variables (endothelial function and clinic BP).

### Outcome measurements

#### Primary outcome

The primary outcome in our study is the change in average 24-h SBP between baseline and 12-week after randomization. Ambulatory blood pressure monitoring (ABPM) will be used to assess the BP level. Participants will wear ABPM kit (Space Labs Medical, ABP Monitor 90,217, Redmond, WA, USA) and maintain their usual activities during the monitoring period, while avoiding strenuous exercise. During a 24-h monitoring period, automatic inflation measurement will be taken every 15 min during daytime (07:00 to 22:59) and every 30 min during night-time (22:59 to 07:00). The 24-h ABPM will be measured at baseline, 12 weeks and 24 weeks after randomization. Participants will be asked to record the time to wake up and go to bed in their self-dairies.

#### Secondary outcomes

The average 24-h DBP, average SBP and average DBP during the daytime and night-time, BP variability, SBP load and DBP load, circadian rhythm of BP, and morning BP surge measured by ABPM will be considered as secondary outcomes.

Endothelial dysfunction is associated with hypertension and its presence correlates with target organ damage. Furthermore, endothelial dysfunction may be both a cause and a consequence of hypertension [41]. Therefore, the change of serum concentrations of Nitric Oxide (NO), endothelin (ET), thromboxane A2(TXA2), vascular endothelial growth factor (VEGF) will be tested to measure the endothelial function using commercial ELISA kits (Bender Med-Systems, Vienna, Austria). Blood samples of participants will be collected at baseline and 12 weeks after randomization.

Home blood pressure monitoring (HBPM) is a self-monitoring tool that can be incorporated into the care for patients with hypertension. Patients will use automated blood pressure monitors (AND, UA-767PBT; Shenzhen, China) to measure BP at home. HBPM will be conducted according to 2019 Chinese Hypertension League guidelines on home blood pressure monitoring [42] during the whole trial. At baseline, patients will measure BP at home every morning (6:00–9:00) and evening (18:00–21:00), 3 times per measurement for 7 days. During intervention and follow-up, patients will measure BP at home every Monday morning (6:00–9:00) and evening (18,00–21:00), 3 times per measurement [42]. Meanwhile, participants will be asked to record BP value in their self-dairies.

Changes in patients’ health-related quality of life will also be assessed according to the Medical Outcomes Study 36-Item Short Form (SF-36) questionnaire Chinese version [43]. This scale contains eight dimensions (physical function, role physical, bodily pain, general health, vitality, social function, role emotional, mental health) and two summary components (physical and mental), with score from 0 to 100. High scores indicate a better quality of life. SF-36 will be measured at baseline and 12 weeks after randomization.

For participants with antihypertensive medication, they will be asked to record the frequency, dosage and types of medication in their self-dairies.

#### Attendance and drop-outs

The attendance of each participants will be checked and recorded in the CRF. Drop-outs from causes such as house moving, onset of severe diseases, or other medical complications are unavoidable, all the details will be recorded in the CRF.

#### Safety monitoring

Adverse events are defined as any unexpected or uncomfortable signs, symptoms, or diseases. These adverse events include falling, fainting and any injury related to exercise. If any adverse events happen during the entire observation period, all the details will be recorded in the CRF.

### Data collection and management

All data will be collected and managed using the Research electronic data capture (REDCap) system [44]. The data will be entered into the REDCap Database by a dependent researcher, then double checked by a second researcher. The statistician will review the database to ensure accurate data collection and correct data export for future analyses. All data will either be kept in a secure and lock-protected location and be backed up in different network drives.

### Investigator training and quality control

We will hold expert consultation meetings to formulate Standard Operating Procedure (SOP) of this study. After that all researchers involved in this study will receive theoretical and practical training courses based on SOP to ensure this trial completely standardized. The training will include how to screen the eligible participants, instruct participants to complete the diary, complete the CRF and assess SF-36 and safety. To ensure the high-quality of this study, quality control assessment will be carried out every month. A specially trained medical officer will inspect the study.

### Statistical analysis

The statistical analysis will be carried out using SPSS statistical package (version 25.0). Data will expressed as mean ± standard deviations. All primary and secondary analyses will be analyzed based on intention-to-treat. Missing data will be filled by Last Observation Carry Forward rules. Demographic characteristics and other baseline values will be described using descriptive statistics. Baseline characteristics of participants will be compared using chi-square test for enumeration data and one-way analysis of variance for continuous variables. For the primary outcome, we will run a comparison between Tai Chi group, walking group and waiting list control group to figure out whether exercise is more effective for this condition. We will then run another comparison between Tai Chi group and walking group to figure out whether Tai Chi is more efficacious for hypertension. The above analysis will be performed using the ANCOVA model, age, pre-intervention BP, duration of hypertension, and family history of hypertension will be used as covariate [45]. Difference between the groups was calculated as mean differences or odds ratios alongside with their 95% confidence intervals. Multiple comparisons between the groups will be adjusted according to the Bonferroni correction method. The correlational analysis of BP and endothelial function factors will be conducted using Chi-square test. We will perform subgroup analyses based on whether patients are on antihypertensive medication to separate the effect size of Tai Chi.

### Patient and public involvement

No patient has been involved in the design, conception and conduction of this trial. All participants are expected to complete the trial by September 2022.

### Ethics and dissemination

This trial will be conducted according to the principles of the Declaration of Helsinki, which has been approved by the regional ethical review committee of traditional Chinese medicine in Sichuan Province in March 2019 (reference number: 2019KL-001). If there is any modification to the protocol which may impact on the conduction of this study, potential benefit of the patient or may affect patient safety, we will draft a formal amendment to the ethical review committee for approval prior to implementation. This clinical trial is also registered with an identifier (NCT04267471) at clinicaltrials.gov in February 2020. Risks and benefits will be explained clearly to the participants, and they will be given enough time to ask questions and decide whether they will participate this trial. The authors intend to publish the findings of the study in peer-reviewed journals.

## Discussion

In our previous systematic review of Tai Chi for hypertension, we found high heterogeneity and poor methodological quality in original RCTs [28]. Among the included trials, only a few trials reported the results of follow-up, the long-term effect of Tai Chi is unclear. Based on the above facts, we hypothesized that: Tai Chi is superior to waiting-list control and walking control in regulating BP of patients with essential hypertension. To test the hypothesis, we designed this trial with a waiting-list group as a blank control group, which will provide an untreated comparison for the Tai Chi group to determine whether it is effective. We will use walking with targeted intensity as active-control group. By comparing with the walking group, whether Tai Chi is better than active control in hypertensive patients will be determined. Our previous study found that intervention of Tai Chi for 12–24 weeks could significantly lower SBP and DBP than intervention < 12 weeks and intervention > 24 weeks [28]. Therefore, we planned 12 weeks of intervention period to determine the effect of Tai Chi.

Existing studies shows that endothelial dysfunction plays an important role in the development of hypertension [46–48]. Regulating endothelial dysfunction has become a significant therapeutic target of hypertension [41, 46, 49]. Aerobic exercise is found to improve endothelial function in hypertensive patients [50, 51]. We assume that Tai Chi reduce BP by regulating endothelial function. Hence, we will additionally investigate the correlation between Tai Chi and endothelial function. In order to achieve high reliability and reproducibility of the results, we will implement more stringent quality control standards. To improve the baseline consistency of participants, we will strictly follow the inclusion and exclusion criteria for participant enrollment. Before formal intervention, included participants will receive standardized Tai Chi training and qualification assessment by experienced coaches to ensure the standardization of Tai Chi movements. During the intervention, we will use fitness tracker to monitor the heart rate of participants to make sure they achieve the target exercise intensity. As for measurement, researchers, Tai Chi coaches and outcome assessors will be separated during the study period. Allocation of participants will be concealed to outcome assessors and data analysts. All researchers will conduct this trial in accordance with SOP. Monthly quality control assessment will be performed to ensure research quality.

We hope the findings of this trial will provide important insight into application of Tai Chi as an effective and acceptable method for hypertensive patients. Successful completion of this proposed study will also contribute to promotion of Tai Chi in the community in the future.

## Data Availability

The datasets used and/or analyzed after completing the current study will be available from the corresponding author by reasonable requests.

## Abbreviations

BP: Blood pressure
CHEP: Canadian Hypertension Education Program
JNC: Joint National Committee
AHA: American Heart Association
SPIRIT: Standard Protocol Items: Recommendations for Interventional Trials
CONSORT: Consolidated Standards of Reporting Trial
SBP: Systolic Blood Pressure
DBP: Diastolic Blood Pressure
CRF: Case report forms
ABPM: Ambulatory blood pressure monitoring
HBPM: Home blood pressure monitoring
SOP: Standard Operating Procedure
SF-36: Medical Outcomes Study 36-Item Short Form
